# A comparison between veterinary small animal general practitioners and emergency practitioners in Australia. Part 2: client-related, work-related, and personal burnout

**DOI:** 10.3389/fvets.2024.1355511

**Published:** 2024-02-28

**Authors:** Kun Li, Erin Mooney, Michelle McArthur, Evelyn Hall, Anne Quain

**Affiliations:** ^1^Sydney School of Veterinary Science, Faculty of Science, The University of Sydney, Camperdown, NSW, Australia; ^2^School of Animal and Veterinary Science, Faculty of Sciences, Engineering and Technology, University of Adelaide, Adelaide, SA, Australia

**Keywords:** burnout, veterinary, mental health, workplace risk-factors, Copenhagen Burnout Inventory

## Abstract

Burnout is reported to be common among veterinarians. However, there is limited research investigating the relationship between specific types of veterinary practice and burnout. A previous study found significant differences in work exposures between veterinary general practitioners (GPs) and emergency practitioners (EPs). The primary aim of this study was to investigate whether Australian veterinary EPs suffer from a higher level of burnout compared to veterinary GPs. The secondary aim of this study was to explore if the previously reported differences between GP and EP groups were positively associated with burnout. An anonymous, online survey incorporating the Copenhagen Burnout Inventory (CBI) was administered to veterinary GPs and EPs practicing in metropolitan regions of Australia. In total, 320 responses were analysed (*n* = 237, 74.2% GPs and *n* = 83, 25.9% EPs). Both groups suffered from moderate levels of burnout, but there were no significant differences in the severity of CBI burnout scores between the two groups. From the multivariable analysis four investigated factors were found to be significantly associated (*p* < 0.05) with the work-related CBI subscale: frequency of finishing work on time; adequate staffing; work satisfaction and seriously considering leaving their principal area of practice. Five factors were significantly associated (*p* < 0.05) with the client-related CBI subscale: position in practice; frequency of client adherence; work satisfaction; frequency of interacting with emotionally distressed clients and seriously considering leaving their principal area of practice. Four factors were significantly associated (*p* < 0.05) with the personal burnout CBI subscale: gender; seriously considering leaving their principal area of practice; frequency of interacting with emotionally distressed clients and the workplace environment. The total burnout score was also significantly associated (*p* < 0.05) with four factors: position in practice, workplace environment, appropriate staffing in the past week and client adherence. Future studies should focus on investigating effective strategies to mitigate these risk factors for both GPs and EPs, to reduce career attrition.

## 1 Introduction

Burnout describes an occupational syndrome deriving from chronically unmanaged work stress and is characterised by three key components: overwhelming exhaustion; increased cynicism and reduced work efficacy (1). It has been previously documented across a range of occupations, including healthcare workers, teachers and other care-giving professions (2–4). The prevalence of burnout among veterinarians, as indicated by studies published in the last 5 years, ranges from 23.0% to 56.9% (5–11). In the 2020 study conducted by Volk et al. (9), which included 2,874 usable responses from US veterinarians, it was revealed that veterinarians reported burnout scores nearly 40% higher than a similar group of physicians and 55% higher than other employed adults.

Numerous individual impacts have been associated with burnout including the development of mental health disorders, cognitive dysfunction, and physical health ailments. A study in human nurses found that respondents who reported significant levels of burnout had a higher likelihood of screening positively for various mental disorders, particularly major depressive disorder (MDD). Respondents with burnout were 43 times more likely to screen positively for MDD (12). Although no similar veterinary studies have been conducted, features of depression and other mental disorders such as post-traumatic stress disorder (PTSD) are often identified together in the same survey groups (6, 8, 13–15). Cognitive impairment is commonly recognised in individuals experiencing burnout, which may manifest as compromised focus, decision making, problem-solving and memory retention (16–19). Physical health disturbances include disrupted sleep patterns in a dose-dependent manner (20), and increased muscle tension resulting in headaches and back pain (21). Chronic activation of the hypothalamus-pituitary-adrenal axis as a result of burnout is associated with an elevated risk of cardiovascular events (22, 23), and increased odds of irritable bowel syndrome (24).

At an organizational level, studies have demonstrated compromise to patient care. A survey conducted among 256 veterinary technicians across four referral hospitals in North America demonstrated a positive association between burnout and medical errors (25). In human healthcare studies, a correlation has been found between burnout and increased healthcare-associated infections (26, 27), with the suggested mediating link to be a breakdown in team efficacy. Individuals experiencing burnout often undergo detachment and reduced emotional control, adversely affecting communication among team members and eroding interpersonal relationships, ultimately leading to a decrease in overall team effectiveness (28, 29). Some individuals with burnout may require extended periods away from work and a percentage may never return to work (30). This results in significant losses through absenteeism (31), attrition, lost income due to position vacancy and the costs associated with staff replacement (32).

Despite studies highlighting the concerns regarding burnout among veterinarians, there is limited research investigating the relationship between the specific type of veterinary practice and burnout. In 2012, a survey study of 27,276 US human physicians reported that those practicing emergency medicine were 3.18 times more likely to experience burnout compared to the general population, while practitioners in family medicine were 1.41 times more likely to experience burnout than the general population (33). This finding was echoed in a more recent meta-analysis highlighting that burnout was most strongly associated with emergency medicine and intensive care physicians, while the lowest association was found among general practitioners (34). Some attribute the higher risk of burnout among emergency medicine physicians to certain working conditions such as schedules that disrupt natural circadian rhythms, workload unpredictability and the exposure to trauma and human suffering (35, 36). In contrast, the American Medical Association’s Organizational Biopsy 2022 found that both emergency medicine (62%) and family medicine (58%) were specialties with the highest percentage of physician burnout (37). They attributed these findings to the rise of Respiratory Syncytial Virus in 2022 overloading emergency rooms and offloading of lower acuity patients to family medicine.

Identifying workplace risk factors predisposing veterinary general practitioners (GPs) and emergency practitioners (EPs) to burnout may enable employers to implement effective strategies to improve workplace conditions and ultimately prevent career attrition. We previously found significant differences in work exposures between GPs and EPs (Li et al., in press). Among respondents, EPs worked a greater variety of shift patterns, including more weekends and public holidays. Veterinary GPs were more prone to performing overtime due to scheduling factors, where EPs were less able to take a meal-break. Additionally, EPs were exposed more frequently to patient death, euthanasia (including financial euthanasia), emotionally distressed clients and had to deliver negative news more often. These findings are echoed in a recent burnout study of emergency veterinarians and technicians that found unmanageable workload, lack of control, insufficient rewards and an unfair allocation of resources to be positively associated with burnout in their group (38). Considering these findings, the primary aim of this study was to investigate whether Australian emergency practitioners (EPs) suffer from a higher level of burnout compared to general practitioners (GPs). A recent Australian study found that unfavourable workplace factors adversely affected veterinarians, despite controlling for individual resilience (39). This highlights the importance of organizational responsibility for addressing modifiable job risk factors. Hence, the secondary aim of this study was to explore if the previously uncovered differences between GP and EP groups were positively associated with burnout.

## 2 Materials and methods

The methodology of this study is described in detail elsewhere (Li et al., in press). Briefly, an anonymous, online survey was administered to veterinary GPs and EPs working in metropolitan regions of Australia between 22nd February 2022 to 22nd June 2022. The survey was built and administered on REDCap (Research Electronic Data Capture), a secure web application for building and managing online surveys, hosted on The University of Sydney’s secure and restricted-access server. The survey consisted of three sections. The first section contained a series of 29 questions (25 main questions and four conditional questions) focused on work-related factors for burnout that may differ between the two groups. In the second section, participants were presented with the three subscales that make up the Copenhagen Burnout Inventory (CBI)—personal burnout, work-related burnout, and client-related burnout (40). The personal burnout subscale is designed to measure the degree of physical and psychological fatigue and exhaustion experienced by an individual (for example, “How often do you feel worn out?”). Work-related burnout is defined as the degree of physical and psychological fatigue and exhaustion one attributes to their work (for example, “Are you exhausted in the morning at the thought of another day at work?”). The subscales were designed so comparison of the personal burnout scale and the work-related burnout scale enables identification of people who attribute fatigue and exhaustion to non-work factors. The client-related burnout subscale measures the degree of physical and psychological fatigue and exhaustion that a respondent perceives to be derived from their work with clients (for example, “Do you feel that you give more than you get back when you work with clients?”). An average score is generated for each subscale, and an overall burnout score is also generated. Scores of 0.0–24.9 indicate no burnout, 25.0–49.9 indicate low burnout, 50.0–74.9 indicate moderate burnout, and 75–100 indicate high to severe burnout. The CBI has been shown to have high internal reliability (Cronbach’s alpha) in previous studies of pharmacists, nurses, and medical doctors (41–43). Response to every item of the CBI was mandatory for participants who wished to submit the survey. The third section of the survey consisted of three demographic questions. The complete survey is provided in Supplementary Table S1.

Power calculation was undertaken prior to recruitment; a sample of 63 respondents in each study group (GPs and EPs) was required to detect a difference of five points on the CBI, assuming standard deviation of 10 units with 80% power and *p* < 0.05. A difference of five points was selected based on the original CBI study comparing 15 human services occupations showing that a difference of five points or more are considered significant (40).

This study was approved by the University of Sydney’s Human Research Ethics Committee (HREC) project number 2022/014.

### 2.1 Statistical analyses

Survey data were downloaded from REDCap into Microsoft Excel^®^ Version 2,301 (Build 16026.20146) to facilitate data cleaning. Where participants selected “other” as their response to a question and a free-form answer was provided, this was re-categorised if it matched one of the options in the drop-down menu. Responses that did not correspond to those in the drop-down menu were retained as “other.” The average scores for all three CBI subscales were calculated for each respondent and the total burnout score was derived from the average of the three subscale scores.

For the questions regarding frequency of interaction with emotionally distressed clients; frequency of delivering negative news and frequency of interacting with adherent clients, the categories “rarely” and “never” were combined into “rarely/never” for the purpose of statistical analysis due to the small number of respondents choosing these categories.

Statistical analyses were conducted in Genstat (Version 18; VSN International, Hemel Hempstead, United Kingdom) and a *p*-value of <0.05 was considered significant for all analyses. Prior to analysis, all four burnout scores (personal, work, client and total) were assessed for normality via the Shapiro–Wilks test. Cronbach’s alpha coefficient was performed for all three CBI subscales and the total score to assess for internal consistency within this survey population. General linear modelling was used to assess the effect of each demographic and work-related factor on each of the four burnout scores (personal, work, client and total). The demographic and work related factors included: GP/EP, sex, age, experience, family composition, position in practice, whether they had seriously considered leaving their current role, hours worked per week, type of shifts, weekend work, public holiday work, having a set roster, having a timely roster notification period, number of unpaid hours per week, frequency of finishing on time, reasons for overtime, quality of meal breaks, staffing, workplace environment, bullying, workplace satisfaction, remuneration, socioeconomic status of clientele, client adherence, frequency of experiencing patient death, euthanasia’s performed per month, frequency of financial euthanasia, frequency of having to deliver negative news, and frequency of dealing with emotionally distressed clients.

A series of univariable linear models were fitted to assess the association of the demographic and work-related factors with each burnout score. Demographic and work-related factors with univariable *p*-values <0.25 were considered for inclusion in the multivariable analyses. A stepwise backwards elimination procedure was used to build each multivariable model, until all terms in the model were significant. Final models were obtained for personal burnout score, work burnout score, client burnout score and total burnout score. All means presented are predicted means with their accompanying standard errors. Post-hoc Tukey’s pairwise analyses were conducted to determine pairwise differences.

## 3 Results

In total, 320 participants completed the CBI in its entirety, with 237 (74.1%) respondents enrolled as GPs and 83 (25.9%) respondents as EPs. The Cronbach’s alpha indicated good to excellent internal consistency for all four scores: work-related burnout 0.752, client-related burnout 0.90, personal burnout 0.895, total burnout 0.808.

**Figure 1:**
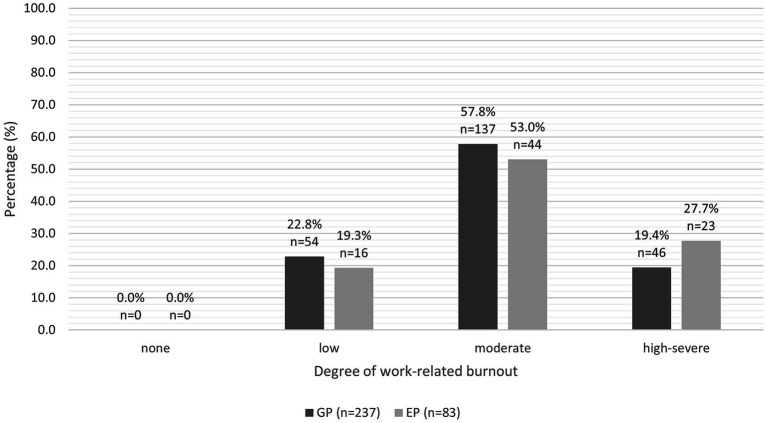
Clustered bar chart for the proportion of GPs and EPs with varying levels of work-related burnout: no burnout (0.0–24.9), low (25.0–49.9), moderate (50.0–74.9), and high-severe (75–100).

**Figure 2:**
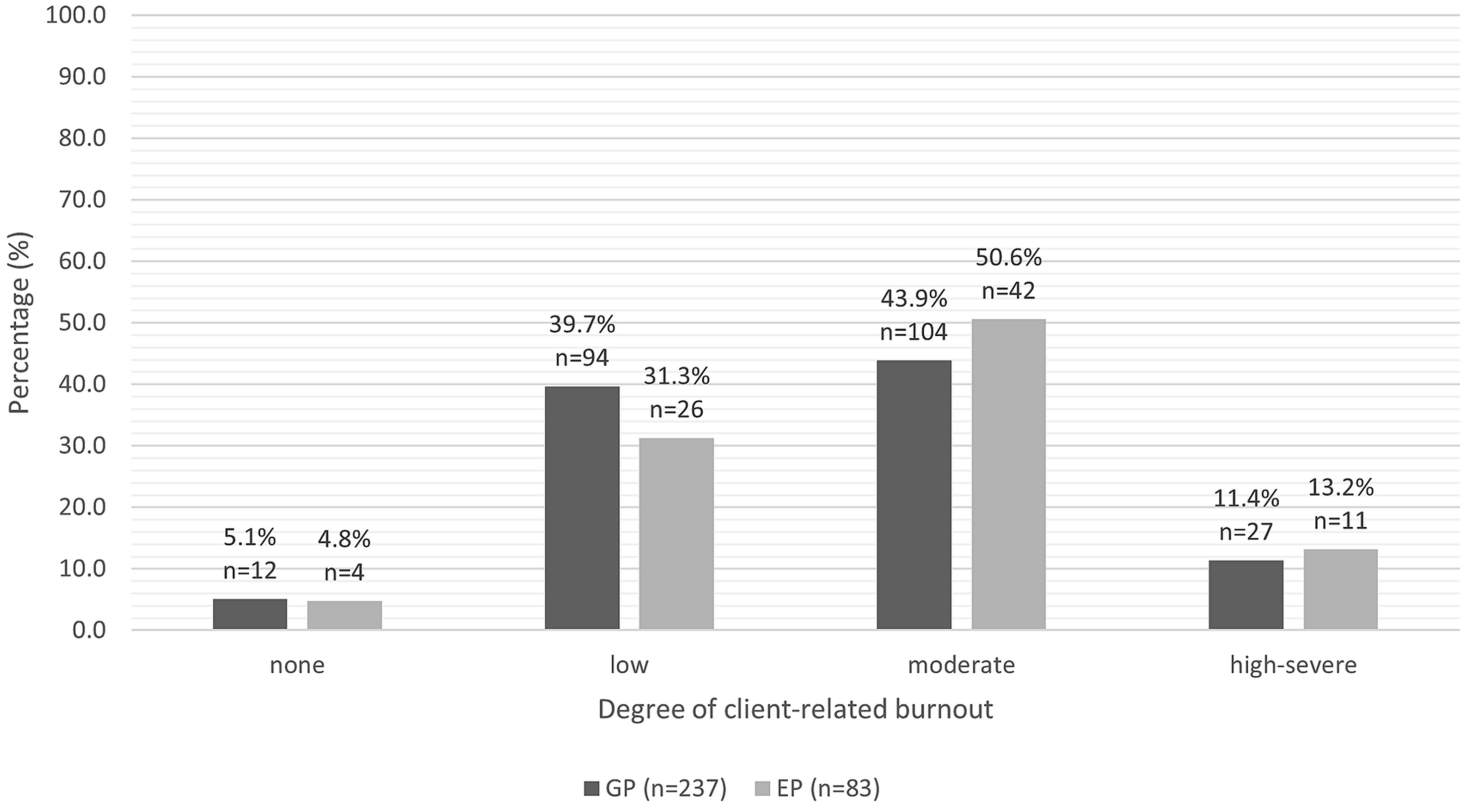
Clustered bar chart for the proportion of GPs and EPs with varying levels of client-related burnout: no burnout (0.0–24.9), low (25.0–49.9), moderate (50.0–74.9), and high-severe (75–100).

**Figure 3:**
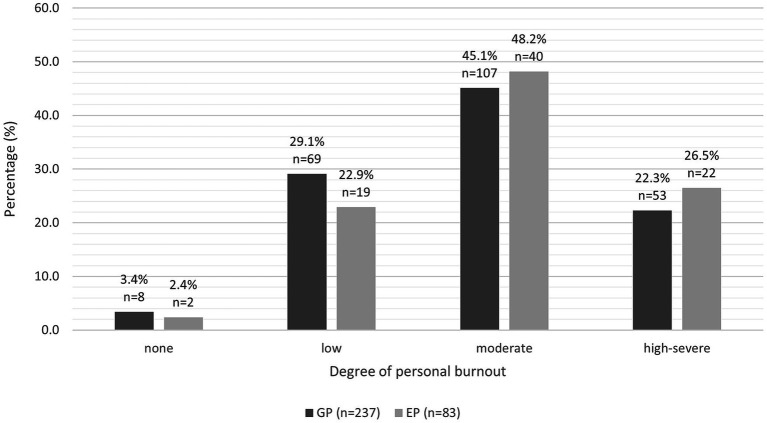
Clustered bar chart for the proportion of GPs and EPs with varying levels of personal burnout: no burnout (0.0–24.9), low (25.0–49.9), moderate (50.0–74.9), and high-severe (75–100).

We found no significant difference in the predicted mean total CBI burnout score, work-related burnout score, personal burnout score or client-related burnout score between the two groups (see Table 1). The predicted mean total CBI burnout score, work-related burnout score and personal burnout score were moderate for both groups. The predicted mean client-related burnout score for GPs was moderate (52.8 ± 2.08), but low for EPs (49.8 ± 1.23). The proportions of GPs and EPs categorised as no burnout, low burnout, moderate burnout or high-severe burnout are illustrated in Figures 1–3.

**Table 1:** Comparison of predicted mean score for general practitioners (GPs, *n* = 237) and emergency practitioners (EPs, *n* = 83) utilising the Copenhagen Burnout Inventory (*n* = 320).

	*p*-value	Principal area of practice	Predicted mean score	Standard error
Work-related burnout	0.152	GP	62.8	1.635
		EP	60.1	0.967
Client-related burnout	0.218	GP	52.8	2.080
		EP	49.8	1.230
Personal burnout	0.156	GP	60.4	2.120
		EP	56.9	1.250
Total burnout	0.111	GP	58.9	1.653
		EP	55.8	0.979

### 3.1 Univariable analysis of demographic and work-related factors

Univariable analysis showed 22 factors were significantly associated with at least one of the three subscale scores or the total CBI score (*p* < 0.05). These factors were: gender, age, years of experience, position in practice, number of hours worked per week, frequency of weekend work, frequency of public holiday work, set and predictable roster pattern, adequate roster notification period, frequency of finishing work on-time, main reason for overtime, meal breaks, socioeconomic status of clientele, frequency of client adherence, frequency of financial euthanasia, frequency of delivering negative news, appropriate staffing in the past week, workplace environment, existence of workplace bullying, work satisfaction, frequency of interacting with emotionally distressed clients, satisfaction with remuneration and whether they had seriously considered leaving their principle area of practice. Additionally, any factors retuning a *p*-value between 0.05 and 0.25 were also considered for inclusion in the subsequent multivariable modelling. The predicted mean of CBI scores, *p*-value and standard error for each investigated factor can be found in Supplementary Tables S2–S5.

### 3.2 Multivariable analysis of demographic and work-related factors

In the multivariable analysis, only four factors were significantly associated with work-related burnout score—frequency of finishing work on time; adequate staffing; work satisfaction and seriously considering leaving the principal area of practice. The client-related burnout score was significantly associated with five factors—position in practice; frequency of client adherence; work satisfaction; frequency of interacting with emotionally distressed clients and seriously considering leaving the principal area of practice. Four factors were significantly associated with the personal burnout score—gender; seriously considering leaving the principal area of practice; frequency of interacting with emotionally distressed clients, and the workplace environment.

The following sections outline the associated significant factors in relation to each CBI subscale, as well as the total score, in more detail.

### 3.3 Work-related burnout

The ability to complete all tasks within rostered hours was significantly associated with the level of work-related burnout (*p* < 0.001). Pairwise comparison (Table 2) showed veterinarians who never finished work on time had a higher predicted mean work-related burnout score (67.6 ± 2.3) compared to veterinarians who always finished on time (53.4 ± 3.5) and to those who could achieve this for most of the time (57.8 ± 1.2).

**Table 2:** Statistically significant factors associated with work-related burnout score—predicted mean, standard errors, and *p*-values.

Factor	*p*-value	Variables	Predicted mean	Standard error
Ability to complete all tasks within rostered hours	<0.001	Always	53.4^b^	3.5
		Majority	57.8^b^	1.2
		Occasionally	61.0^ab^	1.3
		Rarely	62.0^ab^	1.4
		Never	67.6^a^	2.3
Adequate staffing in past week	<0.001	No	63.1	1.1
		Yes	57.7	1.4
Work satisfaction	<0.001	No	63.5	1.5
		Yes	57.2	1.1
Considerations for leaving principal area of practice	<0.001	No	54.0	1.3
		Yes	66.7	1.2

Predicted means with the same subscript are not significantly different after pairwise comparison.

Respondents’ perception that their practice had appropriate staffing over the past week was also significantly associated with work-related burnout (*p* < 0.001). Those who reported that there was not appropriate staffing had a higher predicted mean burnout score (63.1 ± 1.1), compared to those who reported that there was appropriate staffing (57.7 ± 1.4).

Respondents who were satisfied with what they had achieved at work over the past week had a significantly lower burnout score (57.2 ± 1.1) than those who felt dissatisfied (63.5 ± 1.5, *p* < 0.001). Respondents who had seriously considered leaving their principal area of practice within the past year had a significantly higher work-related burnout score (66.7 ± 1.2) than those who had not (54.0 ± 1.3, *p* < 0.001).

### 3.4 Client-related burnout

Veterinarians who always interacted or frequently interacted with emotionally distressed clients had a higher predicted mean client-related burnout score (53.4 ± 4.3 and 50.1 ± 3.3 respectively) compared to veterinarians who rarely/never interacted with distressed clients (43.0 ± 4.2, *p* = 0.002). Those who only interacted with emotionally distressed clients occasionally (43.3 ± 3.3) or rarely/never had low client-related burnout scores (Table 3).

**Table 3:** Statistically significant factors associated with client-related burnout score—predicted mean, standard errors, and *p*-values.

Factor	*p*-value	Variables	Predicted mean	Standard error
Position in practice	0.007	Associate veterinarian	51.6^a^	3.1
		Management	47.8^ab^	4.2
		Owner	43.0^b^	3.7
Frequency of interacting with compliant (adherent) clients in past week	0.007	Always	38.1^a^	6.1
		Majority	48.2^a^	1.8
		Occasionally	56.4^b^	3.1
		Rarely/never	47.0^ab^	9.6
Frequency of interacting with emotionally distressed clients	0.002	Always	53.4^a^	4.3
		Frequently	50.1^a^	3.3
		Occasionally	43.3^ab^	3.3
		Rarely/never	43.0^b^	4.2
Work satisfaction	0.029	No	50.0	3.5
		Yes	45.0	3.1
Considerations for leaving principal area of practice	<0.001	No	42.9	3.3
		Yes	52.0	3.2

Predicted means with the same subscript are not significantly different after pairwise comparison.

Client adherence was also significantly associated with predicted mean client-related burnout scores (*p* = 0.012). Veterinarians working with clients who were only occasionally adherent had the highest predicted mean client-related burnout score (56.4 ± 3.1) compared to veterinarians who work with clients who were always adherent (38.1 ± 6.1) or were adherent for most of the time (48.2 ± 1.8). The group who worked with clients who were rarely/never adherent had a very large standard error of 9.6 associated with the predicted mean of 47.0; indicating a large amount of variation, resulting in non-significant comparisons with the other groups.

The respondent’s position in their practice was significantly associated with their level of client-related burnout (*p* = 0.007). Respondents who identified as associate veterinarians had higher predicted mean client-related burnout (51.6 ± 3.1) when compared to owners (43.0 ± 3.7). Respondents who identified themselves as management had a predicted mean client-related burnout score of 47.8 with a larger standard error of 4.2 and were therefore not significantly associated with the above two groups.

Respondents who were satisfied with what they had achieved at work over the past week had significantly lower client-related burnout score of 45.0 ± 3.1, compared to those who felt dissatisfied (50.0 ± 3.5, *p* = 0.029). Respondents who had seriously considered leaving their principal area of practice within the past year were associated with a significantly higher client-related burnout score (52.0 ± 3.2) compared to those who had not (42.9 ± 3.3, *p* < 0.001).

### 3.5 Personal burnout

Both male and female respondents in this study had moderate levels of personal burnout. Female participants had a significantly higher degree of personal burnout (61.4 ± 2.3, *p* = 0.004) when compared with male participants (54.4 ± 2.9).

The frequency of interacting with emotionally distressed clients was significantly associated with personal burnout (*p* < 0.001). Pairwise comparison (Table 4) showed that when veterinarians dealt frequently with distressed clients, they had a higher predicted mean personal burnout score (61.6 ± 2.1) compared to veterinarians who only interacted with distressed clients occasionally (54.1 ± 2.0) or rarely/never (51.2 ± 3.4).

**Table 4:** Statistically significant factors associated with personal burnout score—predicted mean, standard errors, and *p*-values.

Factor	*p*-value	Variables	Predicted mean	Standard error
Sex	0.004	Female	61.4	2.3
		Male	54.4	2.9
Workplace environment	<0.001	Supportive	51.2^a^	1.5
		Toxic management	60.1^ab^	2.4
		Toxic colleagues	54.2^b^	4.0
		Toxic	61.7^b^	3.9
Frequency of interacting with emotionally distressed clients	<0.001	Always	60.3^ab^	3.4
		Frequently	61.6^a^	2.1
		Occasionally	54.1^b^	2.0
		Rarely/never	51.2^b^	3.4
Considerations for leaving principal area of practice	<0.001	No	51.5	2.6
		Yes	64.4	2.4

Predicted means with the same subscript are not significantly different after pairwise comparison.

Workplace culture was also significantly associated with the level of personal burnout (*p* < 0.001). Pairwise comparison showed veterinarians working in environments with supportive colleagues and management teams had a lower predicted mean personal burnout score (51.2 ± 1.5) when compared to veterinarians who reported working in environments with toxic colleagues and management teams (61.7 ± 3.9) and toxic colleagues only (54.2 ± 4.0). The predicted mean for the group who worked with toxic management only was 60.1 ± 2.4, however this group was not found to be significantly different when compared to the other groups.

Respondents who had seriously considered leaving their principal area of practice within the past year had a significantly higher predicted mean personal burnout score (64.4 ± 2.4) compared to those who had not, 51.5 ± 2.7 (*p* < 0.001).

### 3.6 Total burnout score

Four of the demographic and work-related factors were significantly associated with total burnout: position in practice, workplace environment, appropriate staffing in the past week and client adherence (Table 5). The only notable difference in pairwise comparison between the predicted mean total burnout score and its subscales was related to client adherence. Veterinarians who worked with compliant clients only occasionally, recorded a higher predicted mean total burnout score (63.8 ± 2.6) compared to veterinarians who work with compliant clients for the majority of the time (55.6 ± 1.7, *p* = 0.007). The groups of “always” and “rarely/never” were not found to be significantly different to the other groups.

**Table 5:** Statistically significant factors associated with total burnout score—predicted mean, standard errors, and *p*-values.

Factor	*p*-value	Variables	Predicted mean	Standard error
Position in practice	0.019	Associate veterinarian	59.9^a^	2.6
		Management	59.5^ab^	3.6
		Owner	53.5^b^	3.2
Adequate staffing in past week	<0.001	No	60.8	2.8
		Yes	54.5	2.9
Workplace environment	0.003	Supportive	53.8^a^	2.5
		Toxic management	59.6^ab^	3.1
		Toxic colleagues	63.9^b^	4.1
		Toxic	63.2^b^	3.8
Frequency of interacting with compliant (adherent) clients in past week	0.007	Always	55.4^ab^	5.1
		Majority	55.6^a^	1.7
		Occasionally	63.8^b^	2.6
		Rarely/never	55.7^ab^	8.0

Predicted means with the same subscript are not significantly different after pairwise comparison.

## 4 Discussion

Both GPs and EPs displayed a moderate level of burnout, although there was no significant difference in the predicted mean total CBI burnout scores between veterinary GPs and EPs. This finding was unexpected and could be due to several factors. As we have previously shown, there are some fundamental commonalities between GPs and EPs. Both groups provide primary care for companion animals and the majority of both workforces are made up of non-specialist veterinarians. The contributing factors found to be associated with total burnout (position in practice, staffing adequacy, workplace culture, client adherence) were not points of significant difference between the two groups. Both groups are exposed to the same wider Australian veterinary workforce environment and associated factors, such as the generalized workforce shortages (44) and increased pressures during the COVID-19 pandemic (45, 46). We could not control for the influence of these wider workforce issues, which could have blunted potential differences between the level of burnout of the two groups. In our previous study, we found significant differences in the demographic and work factors between GPs and EPs (Li et al., in press). Some of those differences were significant in contributing to total burnout on univariable analysis. However, none remained significant in the multivariable analysis. This highlights the multifactorial nature of burnout.

The association between perceived inadequate staffing and higher total and work-related burnout scores was expected as inadequate staffing contributes to increased workload. In 2021, during the pandemic, veterinarians were added to the Australian priority skilled migration list, reflecting the widespread workforce shortages (47). The veterinary workforce shortage is reflected in the 2021 AVA Workforce Survey, which showed 77.5% of respondents were working in a practice that was advertising a position for a veterinarian (44). Staff shortages were exacerbated by the COVID-19 pandemic in two ways. Firstly, due to public health orders, veterinary team members had to modify their working practices. In some instances this included working in split teams to ensure continuity of veterinary care if one team were exposed and thus required to isolate (45). Additionally, veterinarians were less inclined to attend work with influenza-like illness (48). Nonetheless, 75% of veterinarians indicated that they would still attend work with a “dry cough” as they felt no coverage was available (48). Sick presenteeism has been shown to lead to decreased productivity at work and compromised patient care in human healthcare workers (49, 50). Thus both sickness absenteeism and presenteeism likely contributed to increased staff shortages and relative increase in caseload. Another reason was the documented boom in pet ownership during the pandemic, which would have increased caseload and lead to relative staff shortages (51, 52). The workforce shortages continue, with one Australian State Government (New South Wales) currently running an inquiry into the veterinary workforce shortage (53). With the current workforce shortages in Australia, it is difficult for individual employers to address this problem directly. A strategy with more rapid effect, could be increasing support staff to aid in alleviating veterinarians of tasks that can be performed by nurses or technicians or administrative staff.

In our study, practice owners showed lower total burnout scores than associate veterinarians. This is in agreement to prior veterinary studies in Germany (54) and the US (55). The characteristics of the practice owner role gives these veterinarians greater decision latitude and autonomy. In a recent Australian study, 61.7% of veterinarians indicated that they were given little or no control over the structure of their workday (39). A lack of control has been shown to contribute to job strain, anxiety, burnout and depression, especially in high-demand jobs (38, 39, 56, 57). Conversely, encouraging increased participation in decision-making improved psychological health and absenteeism (58, 59). This could explain the increasing trend of veterinarians moving to locum work in recent years to gain more autonomy over their work structure and commitments (44, 60). Locum veterinarians also enjoy the added benefit of higher salaries compared to employees (61). This trend is concerning in the face of workforce shortages, as it can create greater instability in the workforce.

Being a practice owner was also associated with lower levels of client-related burnout. This could be attributed to less exposure to clients due to greater managerial duties. While practice owners are more likely to be involved in resolving challenging client complaints, it is possible that this added stress is outweighed by increased discretion in resolving such matters, for example, being able to offer a discount. If associate veterinarians are expected to participate in the complaint handling process, then a recommendation can be made to provide employees with sufficient level of delegated authority to enable complaint resolution and to have clear communication on the pathway of complaint escalation in the workplace (62).

A low level of client adherence was associated with higher total and client-related burnout. Veterinarians are often faced with ethically challenging situations. A 2018 study found that 85% of respondents “often” or “sometimes” had different and conflicting opinions to clients about the care of their companion animals, and 79% indicated that not being able to provide appropriate care caused moderate to severe distress (63). This result was echoed in a global survey of veterinary team members on ethically challenging situations. It found that “conflict between the interests of clients and the interests of their animals” was one of the most encountered ethically challenging situations and the most stressful for veterinarians (45). Both studies found that veterinarians were inclined to deal with these situations by discussing them with colleagues and seeking professional reassurance that their decision was correct. This requires a psychologically safe workplace culture and would be problematic in a toxic workplace. One study reported that 71% of respondents have received no conflict resolution training to deal with these complex situations (63). One of the keys to addressing poor client adherence is to understand the rationale behind differences in opinion by optimizing communication skills (64), as poor adherence can be the result of different understanding of an animal’s medical condition, misaligned expectations, mistrust or financial or practical constraints. Providing training opportunities in ethics, communication and conflict resolution may better equip veterinarians to manage these situations (45, 65). Setting up regular workplace ethics rounds may provide veterinarians with a psychologically safe space to debrief and gain support from their colleagues (66, 67).

Frequent interaction with emotionally distressed clients was associated with higher client-related and personal burnout scores raising concerns that this type of client interaction may also carry a negative impact to the veterinarian’s personal life. A recent Burden Transfer Inventory (BTI) study found a positive correlation between the frequency of veterinarians experiencing client grief and burnout, likely due to compassion fatigue (68, 69). The extent of the effect of the BTI item on the individual was the main predictor, suggesting personal strategies may help to fortify an individual against burnout (68). The substantial emotional burden in guiding clients through challenging medical decisions in the face of an unfavourable diagnosis is sometimes compounded by the veterinarian’s personal emotional investment in their patients (8, 70). This dual layer of emotional strain increases the likelihood of compassion fatigue, leading to heightened emotional exhaustion. Frequent exposures to emotionally distressed clients was one of two burnout-associated factors that was found to be significantly different between GP and EP groups in the authors’ previous study (Li et al., in press), with EPs found to have more frequent exposures than GPs.

“Never” being able to complete all required work within their rostered time was associated with higher work-related burnout scores compared to respondents who were able to do so “always” or for the “majority of the time.” This finding aligns with previous studies that showed unmanageable workload was positively correlated with burnout (38, 56). Excessive workload causes spill-over negative effects such as increased sickness presenteeism and inability of veterinarians to take meal-breaks (39, 48) (Li et al., in press). Workload was another point of difference identified in the previous comparison between GP and EP groups. In the previous study, the authors found GPs could “rarely” finish on time compared to EPs who could finish on time for the “majority of the time.” In this study no significant difference was found between these two categories due to high variations in burnout scores.

There are currently 8 universities in Australasia—7 in Australia and one in New Zealand—offering veterinary degrees. The number of veterinary degree completions has risen from under 500 *per annum* in 2008, to a projected estimate of over 900 by 2025, of whom approximately one-fifth will be international students (71). Thus, it is unlikely that workforce shortages are due to a lack of new graduates, but rather due to attrition. In the AVA Federal Government Pre-Budget Submission 2023, the peak body characterised one of the reasons for attrition to be “high rates of burnout, stress, and negative mental health outcomes” (72). The results of this current study add evidence to this with higher burnout scores recorded in all three CBI subscales in GPs and EPs who had seriously considered leaving their principal area of practice. We found that in this respondent population 60.2%, had considered leaving their principal area of practice within the past year, of which 31.3% were thinking of leaving the profession. It is of vital importance to focus on retention strategies within the veterinary industry by building work satisfaction and targeting modifiable work-related factors to reduce burnout from an organizational level (39).

### 4.1 What can employers of general practice and emergency veterinarians do to help decrease burnout?

In the short term, employers may be able to increase support staff numbers to reduce workload of veterinarians (73). In the longer term, registration of veterinary nurses and technicians in Australia with clear guidelines regarding legal responsibilities would aid in alleviating workload and reduce the burden of responsibility on veterinarians. To give veterinarians more decision latitude and better manage their workloads, employers could actively seek participation from the veterinarian in organizing their schedules. This will ensure that GP veterinarians are afforded appropriate meal-breaks and protected time for hospital patients, diagnostic tests, follow-up phone calls and record keeping.

Due to the nature of emergency work, scheduling is less modifiable. However, employers can look to roster more effectively to ensure adequate overlap to allow meal breaks and to allow EPs appropriate time to complete diagnostics, call-backs, patient handovers, and records towards the end of their shift. For veterinarians working a varied roster, employers should provide a roster notification period of at least 4 weeks to meet the Australian industry award (74), but ideally longer (Li et al., in press) to enable veterinarians to plan their schedules outside of work. More research is required to determine what is an appropriate workload (reflected by wait times and acuity levels) for veterinary emergency centers. If severe workforce shortages continue, then local authorities such as veterinary registration boards should develop appropriate care diversion protocols and infrastructure to allow increased communication and collaboration between veterinary hospitals to help with resource distribution (46).

Client-related burnout can be addressed by improving client adherence and developing healthy self-care habits to prevent compassion fatigue (75). Communication is key in developing trust and understanding the client’s concerns and constraints that may be preventing adherence to veterinary advice. Communication workshops on reflective listening and conflict management may be beneficial (45). Importantly, veterinarians require time for these conversations, therefore appropriate scheduling of consultation time remains paramount. As veterinarians are often faced with ethically challenging situations, employers could offer continuing education workshops on applying ethical frameworks in decision making and also self-care workshops to equip employees with tools to combat compassion fatigue (76). Management can also help by facilitating regularly scheduled team debriefing sessions or ethics rounds that can help with alleviating moral distress and increasing teamwork (67).

Fostering a healthy workplace culture is important in the success of these interventions as shame and fear of judgment from colleagues are the main barriers in seeking help (77). We previously found that workplace bullying was prevalent in the workplace for this group of respondents (Li et al. in press). Every team member has a personal responsibility to provide a blame-free, psychologically safe environment to allow adequate debriefing and to promote a culture where errors are considered an opportunity to learn. The leadership group has a strong influence over workplace culture through both policy making and practices to address complaints of bullying (78). In environments where management teams are described as “toxic” there is often a feeling of injustice among employees, derived in part from a lack of mutual understanding of unique stressors and damaged trust from inconsistent communication lacking in transparency (79–81). Transparency in communication is key in developing trust. It is also important to actively listen, invite employees to voice concerns and ensure concerns are dealt with justly and appropriately in a timely manner (82).

In this survey respondent group, 52.0% were dissatisfied with their remuneration (Li et al., in press), but this was not associated with increased burnout. However, dissatisfaction with remuneration could still represent a factor for considering leaving the profession. Previous studies identified low salary as an important factor contributing to attrition, as did the AVA Federal Government Pre-Budget Submission 2023 (61, 72, 83). The Fair Work Ombudsman should look to update the minimum veterinary service awards to reflect the current market trends. In the meantime, employers can consult the AVA Workforce Survey Salary Integration Report 2021 (84).

### 4.2 Limitations

We elected to employ the Copenhagen Burnout Inventory (CBI) as the psychological instrument in our study due to its high validity and reliability (85, 86). Within our respondent group it demonstrated good to excellent internal reliability for all four burnout scores investigated, which is a key strength of our study. The CBI offers reliable comparison of degree of burnout, making it ideal for our primary research objective. The three subscales (work-related, client-related, and personal burnout) were congruent with the working context of veterinarians in a clinical setting. We do recognise its shortcoming in being a continuous measure of burnout severity in its design (40). Categorical cut-offs were assigned based on a previous study in Australian midwives due to its similar geographical context (87). We recognise that this cut-off was assigned by the rationale of numerical designation on the original five-point Likert scale of the instrument—100 (always), 75 (often), 50 (sometimes), 25 (seldom) and 0 (never/almost never) (40), however it has not been validated. In our literature search, no validation studies have been performed on categorical cut-offs for the CBI. This is a common limitation of the current burnout measures available (88). Some preliminary work on validation of cut-off points have been performed on the Maslach Burnout Inventory (MBI) (89, 90) and Burnout Assessment Tool (BAT) (88). The MBI was not utilised for this study due to the criticisms surrounding this instrument (40, 86, 91, 92) and limitations in funding. The BAT cut-off validation study was performed after the completion of this study, so could not be included during study planning.

A response rate could not be calculated as it was not possible to quantify the population of people who saw the survey but did not click on the survey link. Recruitment for this survey was performed through voluntary participation and hence self-selection. This often introduces a non-response bias as individuals who feel more strongly about burnout may be more likely to respond and hence may not truly reflect the general veterinary GP and EP population in Australia. Mean CBI burnout scores were compared in this study, however large variations observed for several investigated variables resulted in non-significant results, where a larger sample size may have reduced the variation and allowed for clearer differentiation. Lastly, the CBI questions were presented under one section with unidirectional options (never to always). However, it is recommended to have some questions in reverse direction options (always to never) to avoid stereotyped response patterns (40). Additionally, it is possible that some terms or phrases in the survey, such as “toxic workplace” or “collegial/supportive workplace” could have been interpreted differently by respondents or may have been leading. We piloted the survey with a diverse cohort to minimise leading questions but acknowledge that the phrasing could be a source of bias in our results.

An inherent limitation of cross-sectional survey studies is the lack of ability to infer causality as not all variables can be controlled for. It was also not possible to determine the directionality of associated variables for some of the risk factors investigated. For example, never being able to finish work on time was associated with higher work-related burnout scores. An alternative interpretation could be that respondents with higher burnout scores are less efficient and hence can never finish work on time.

The COVID-19 pandemic created pressures on the veterinary workforce, for example through public health orders that restricted movement, mandated physical distancing and enforcing sickness absenteeism, exacerbating staff shortages. Veterinary team members were forced to work differently. Thus, it would be of value to repeat this study on the same population after the pandemic to eliminate pandemic associated factors. It would also be of interest to repeat this study internationally to investigate if common contributors to burnout are shared globally. Based on the findings of this study, some mitigating strategies are recommended to employers. Further veterinary research is required to explore the potential for success of these strategies in the veterinary profession.

## 5 Conclusion

Australian veterinary GPs and EPs suffer from moderate levels of burnout. There were no significant differences in the severity of CBI burnout scores between the two groups. This study confirms that burnout is associated with seriously considering leaving the principal area of practice suggesting that burnout contributes to veterinary workforce shortages. Out of the work-related factors investigated, we found that staffing shortages, toxic workplace culture, a low degree of client adherence, increased frequency of interacting with emotionally distressed clients, never being able to finish work on time, and being an associate veterinarian were associated with higher burnout scores. Being satisfied with one’s achievements at work was significantly associated with lower work-related and client-related burnout scores. Future studies should focus on investigating effective strategies to mitigate these risk factors for both GPs and EPs.

## Data availability statement

The datasets presented in this article are not readily available because conditions of the University of Sydney Human Research Ethics Committee (HREC) approval. Requests to access the datasets should be directed to anne.quain@sydney.edu.au.

## Ethics statement

The studies involving humans were approved by the University of Sydney Human Research Ethics Committee (HREC). The studies were conducted in accordance with the local legislation and institutional requirements. The participants provided their written informed consent to participate in this study.

## Author contributions

KL: Conceptualization, Data curation, Formal analysis, Investigation, Methodology, Project administration, Writing – original draft, Writing – review & editing. EM: Conceptualization, Supervision, Writing – review & editing. MM: Methodology, Supervision, Writing – review & editing. EH: Data curation, Methodology, Software, Writing – original draft. AQ: Conceptualization, Funding acquisition, Investigation, Methodology, Resources, Supervision, Writing – review & editing.
