# Supplementary material for: A comparison between veterinary small animal general practitioners and emergency practitioners in Australia. Part 2: client-related, work-related, and personal burnout
Source: Front Vet Sci. 2024 Feb 28;11:1355511. doi: 10.3389/fvets.2024.1355511 (PMC10932987; doi:10.3389/fvets.2024.1355511)
Supplement: Supplementary file 1 [file Table_1.pdf]

Supplementary Material

Supplementary Table 1. Survey Questions - A survey study of burnout and risk factors in Australian emergency and general practice veterinarians.

**Questions**	**Options**
I have read the participant information sheet. I am over the age of 18 and consent to participate in this study.	Yes<br>No
I am a small animal veterinarian, who is currently practicing in a metropolitan area within Australia. I am currently working in either emergency or general practice. Definition of metropolitan area according to the Rural, Remote and Metropolitan Areas Classification (RRMA) 1991: an urban centre with a population of 100 000 or more. If you are unsure whether you are working in a metropolitan area, you can check your RRMA classification by searching your suburb on: https://www.health.gov.au/resources/apps-and-tools/health-workforce-locator/health-workforce-locator	Yes<br>No
**Part 1/3 – Work Related Questions**	
What is your principal area of practice?<br>*Place of work where you have spent more than 60% of your time in the last 3 months.	Small animal general practice<br>Small animal emergency practice
How long have you worked in your principal area of practice (GP / emergency)?<br>*In years (e.g., 6 months = 0.5 years)	(Free text response)
What is your position in your practice?	Associate veterinarian<br>Management<br>Practice owner
On average, how many hours do you work per week?<br>*Include all paid and unpaid overtime	Less than 35 hours per week<br>35 - 40 hours per week<br>40 - 50 hours per week<br>50 - 60 hours per week<br>Greater than 60 hours per week
Which of the following best describe your shift pattern in the past month?	Only day shifts (work hours largely fall between 7am - 7pm)<br>Only night shifts (work hours largely fall between 7pm - 7am)<br>Majority day shifts (more than 70%)<br>Majority night shifts (more than 70%)<br>None of the above (less than 70% of either day or night shift)
How often do you work on the weekend?	At least one day every weekend<br>1 in 2 weekends<br>1 in 3 weekends

	1 in 4 weekends<br>Infrequently (less than 1 in 4 weekends)<br>I never work on the weekend
Which of the following statements best describe your work schedule on a public holiday?<br>*Please answer with regard to the public holidays observed in the state/territory in which you work	I am not required to work on a public holiday.<br>I am able to choose if I want to work on a public holiday.<br>I am required to work less than 50% of public holidays.<br>I am required to work 50% or more public holidays.<br>I am required to work every public holiday
My roster pattern is set and predictable.	Yes<br>No
I receive my roster well enough in advance to be able to plan my life outside of work.	Yes<br>No
In relation to the previous question, on average how many weeks in advance do you receive your roster?<br>(In weeks)	(Free text response)
On average, how many unpaid hours do you work per week?	Less than 1 hour<br>1 - 3 hours<br>4 - 6 hours<br>7 - 10 hours<br>Greater than 10 hours
How frequently are you able to finish all required work within your rostered shift time?	Always<br>Majority of the time.<br>Occasionally<br>Rarely<br>Never
What is the main contributing factor to not finishing on time?	Scheduling factors<br>Inappropriate staffing or short staffing<br>Personal reasons<br>Equipment/software inadequacies<br>Pressure to fit in walk-in consults<br>Other
If other, please specify.	(Free text response)
In the past week, the following statement best describes my meal breaks.<br>*Please answer based on what happened for the majority of the week.	Unable to take a meal break (< 30 minutes)<br>Meal break 30min - 1 hour, interrupted by work<br>Meal break 30min - 1 hour, uninterrupted<br>Meal break greater than 1 hour, interrupted by work<br>Meal break greater than 1 hour, uninterrupted

In regards to your current place of work, how would you describe the socioeconomic situation of the majority of your clientele?	Low income and pensioners<br>Middle income<br>Upper-middle income<br>Affluent income<br>Diverse (even mix of all of the above)
Think back to the past week, how often are your clients receptive and compliant with your diagnostic and treatment recommendations?	Always<br>Majority of the time<br>Occasionally<br>Rarely<br>Never
How many times did you experience patient death (non-euthanasia) in the past month?	None<br>1-3 times<br>4-6 times<br>Greater than 6 times<br>Do not recall
How many euthanasias were you requested to perform in the past month?	None<br>1-3<br>4-6<br>7-10<br>Greater than 10
In relation to the previous question, how many euthanasias were you requested to perform where the primary reason was due to financial limitations?	Always<br>Frequently<br>Occasionally<br>Rarely<br>Never
In the past week, how often were you required to deliver negative news* to clients regarding their pet's health?<br>*negative news can be serious illness, life-threatening conditions, terminal illness or recommendation for euthanasia.	Always<br>Frequently<br>Occasionally<br>Rarely<br>Never
In the past week, I feel that my practice was appropriately staffed on most days of the week.	Yes<br>No
Which of the following statements best describe your current workplace environment?	Both my colleagues and the management team are supportive and collegial.<br>My colleagues are supportive and collegial, but I am pressured by a toxic management team.<br>My colleagues are toxic, but my management team is supportive.<br>Both my colleagues and the management team contributes to a toxic workplace environment.
In your current place of work, have you experienced or witnessed workplace bullying?	Experienced workplace bullying<br>Witnessed workplace bullying<br>No, did not experience or witness workplace bullying

In the past week, I feel satisfied with what I have achieved at work.	Yes<br>No
In the past week, how often were you interacting with emotionally distressed clients?<br>*For example: anxious, sad or angry clients.	Always<br>Frequently<br>Occasionally<br>Rarely<br>Never
I am satisfied with my renumeration for the work that is required of me.	Yes<br>No
In the past year, have you seriously considered leaving your principal area of practice (GP/Emergency)?	Yes<br>No
After leaving your current role, what type of work will you consider transitioning into?	Another type of clinical role in veterinary medicine<br>Pursue further training or specialisation within veterinary medicine<br>Non-clinical role, but related to veterinary medicine<br>Leaving the veterinary medicine profession<br>Undecided
**Part 2/3 – Burnout Measurement**<br>The following three sets of similar questions are in relation to how you feel about your work, client interactions and personal life.	
**Work burnout**	
Do you feel worn out at the end of the working day?<br>Are you exhausted in the morning at the thought of another day at work?<br>Do you feel that every working hour is tiring for you?<br>Do you have enough energy for family and friends during leisure time?<br>Is your work emotionally exhausting?<br>Does your work frustrate you?<br>Do you feel burnt out because of your work?	Never<br>Seldom<br>Sometimes<br>Often<br>Always
**Client burnout**	
Do you find it hard to work with clients?<br>Does it drain your energy to work with clients?<br>Do you find it frustrating to work with clients?<br>Do you feel that you give more than you get back when you work with clients?<br>Are you tired of working with clients?<br>Do you sometimes wonder how long you will be able to continue working with clients?	Never<br>Seldom<br>Sometimes<br>Often<br>Always
**Personal burnout**	
How often do you feel tired?<br>How often are you physically exhausted?<br>How often are you emotionally exhausted?	Never<br>Seldom<br>Sometimes

How often do you think: "I can't take it anymore"?<br>How often do you feel worn out?<br>How often do you feel weak and susceptible to illness?	Often<br>Always
**Part 3/3 – Demographic characteristics**	
Which gender do you identify with the most?	Female<br>Male<br>Other
What is your age?<br>(In years)	(Free text response)
What is your household make up?	Single, no dependent children<br>Single, with dependent children<br>Married / de-facto relationship, no dependent children<br>Married / de-facto relationship, with dependent children
Thank you for answering the survey questions. If you feel that there are reasons for burnout that is not captured in this survey or if you have any general feedback, please comment in the box below:	(Free text response)
If you are interested in receiving a copy of the final results from this study, please email: kun.li@sydney.edu.au<br>If any of the questions in this survey has evoked feelings of distress, we would recommend seeking assistance from professional organisations or a health professional, including:<br>• Beyond Blue Support Services - Ph: 1300 22 4636<br>• Lifeline Australian - Ph: 13 11 14<br>• Your doctor<br>I understand that by submitting my responses I am consenting for the information I have entered to be used for research purposes.	

Supplementary Table 2. Univariable analysis results for Copenhagen Burnout Inventory – personal burnout score. This table presents the P-value, predicted mean and standard error for each investigated factor and associated variable.

Factor	P-value	Variables	Predicted mean	Standard error
Sex	<0.001	Female	60.0	1.18
		Male	48.7	2.42
Age (years)	0.0219	<30	60.3	2.82
		30-40	61.5	1.77
		40-50	54.5	2.17
		50+	54.5	2.17
Family composition	0.081	Married with dependent children	55.6	1.90
		Married with no dependents	56.4	1.70
		Single with dependent children	61.1	5.55
		Single with no dependents	62.3	2.15
Years of experience in principal practice	0.013	<5	62.1	2.06
		5 to 10	60.1	2.44
		10 to 20	56.4	2.16
		20+	53.1	2.04
Considering leaving principal practice in the past year	<0.001	No	46.8	1.54
		Yes	65.2	1.24
Position in practice	0.010	Associate Veterinarian	59.2	1.23
		Management	59.6	3.68
		Owner	50.2	2.73
Hours worked per week	0.352	< 35 hours	55.9	1.93
		35-40 hours	57	1.85
		40-50 hours	58.7	2.25
		50-60 hours	63.9	3.95
		> 60 hours	62.5	5.36
Shift pattern	0.105	> 70% night shifts	58.8	4.30
		> 70% day shifts	61.1	2.59
		Only day shifts	55.9	1.31
		Only night shifts	57.7	5.55
		A combination of day and night shifts	62.2	4.97
Weekend work	0.222	1 in 2	60.9	1.85
		1 in 3	55.9	2.49
		1 in 4	56.8	2.73
		1 day per weekend	59.7	2.98
		Infrequently (< 1 in 4)	52.5	3.41
		Never	54.8	3.78
	0.017	Can choose	55.4	2.88

Public holiday working requirement		Every public holiday	67.9	5.29
		Not required	54.9	1.56
		<50% of public holidays	59.4	2.53
		>50% of public holidays	63.5	2.55
Set roster pattern	0.090	No	60.6	2.01
		Yes	56.6	1.28
Timely roster notification	<0.001	No	69.5	2.79
		Yes	53.6	2.36
Average number of unpaid work hours per week	0.107	< 1	56.2	1.58
		1-3	56.8	2.23
		4-6	60.2	2.78
		7-10	54.2	4.68
		>10	65.8	3.52
Able to finish on time (workload)	<0.001	Never	71.6	3.65
		Rarely	61.4	2.14
		Occasionally	56.8	1.95
		Majority	54.3	1.74
		Always	43.2	5.62
Main reason for overtime	0.007	Scheduling	57.0	1.69
		Short staff	60.5	1.86
		Personal	36.1	6.33
		Walk-ins	58.4	2.47
		Other	59.0	4.25
Able to have meal break	<0.001	No	62.8	1.57
		Interrupted meal break	55.0	1.62
		Meal break without interruption	49.8	2.87
Adequate staffing in the past week	<0.001	No	61.7	1.32
		Yes	51.4	1.71
Workplace environment	<0.001	Supportive	53.3	1.24
		Toxic colleagues only	60.6	4.29
		Toxic	70.8	4.07
		Toxic management only	68.0	2.24
Bullying	<0.001	Experienced	65.1	2.41
		None	53.6	1.45
		Witnessed	60.7	1.99
Work satisfaction	<0.001	No	68.3	2.06
		Yes	54.3	1.19
Renumeration satisfaction	<0.001	No	62.7	1.45
		Yes	52.4	1.51
Socioeconomic status of clients	0.01	Affluent	64.3	3.48
		Diverse	60.6	1.88
		Low income	60.0	3.60
		Middle income	56.7	2.11
		Upper-middle	52.0	2.17

Frequency of interacting with adherent clients in the past week	0.02	Always	55.7	6.78
		Majority	56.6	1.17
		Occasionally	66.5	3.03
		Rarely/never	56.9	11.06
Frequency of encountering patient death in the past month	0.249	None	55.6	1.63
		1-3	59.9	1.64
		4-6	63.3	4.83
		> 6	53.9	4.83
		Do not recall	57.8	6.83
Frequency of euthanasia in the past month	0.150	None	61.1	5.56
		1-3	53.4	2.03
		4-6	59.0	1.87
		7-10	60.1	2.53
		> 10	59.6	2.62
Frequency of economic euthanasia in the past month	0.0213	Frequently	65.3	4.18
		Occasionally	59.5	1.81
		Rarely	58.3	1.79
		Never	52.4	2.26
Frequency of delivering negative news in the past week	0.060	Always	60.6	4.53
		Frequently	59.6	1.56
		Occasionally	56.6	1.65
		Rarely/never	46.9	4.96
Frequency of interacting with emotionally distressed clients in the past week	<0.001	Always	61.8	3.50
		Frequently	63.9	1.65
		Occasionally	53.9	1.57
		Rarely/never	45.7	3.50

Supplementary Table 3. Univariable analysis results for Copenhagen Burnout Inventory – work-related burnout score. This table presents the P-value, predicted mean and standard error for each investigated factor and associated variable.

Factor	P value	Variables	Predicted mean	Standard error
Sex	<0.001	Female	62.2	0.91
		Male	54.7	1.88
Age (years)	0.032	<30	64.1	2.16
		30-40	63.6	1.36
		40-50	58.7	1.66
		50+	56.8	1.66
Family composition	0.420	Married with dependent children	59.5	1.48
		Married with no dependents	60.2	1.33
		Single with dependent children	61.6	4.32
		Single with no dependents	63.1	1.67
Years of experience in principal practice	0.010	<5	64.4	1.58
		5 to 10	61.8	1.88
		10 to 20	59.8	1.66
		20+	57.1	1.57
Considering leaving principal practice in the past year	<0.001	No	61.3	1.13
		Yes	67.1	0.92
Position in practice	<0.001	Associate Veterinarian	62.7	0.92
		Management	59.3	2.78
		Owner	51.8	2.06
Hours worked per week	0.471	< 35 hours	60.2	1.49
		35-40 hours	60.0	1.43
		40-50 hours	61.9	1.73
		50-60 hours	56.0	3.05
		> 60 hours	56.9	4.14
Shift pattern	0.105	> 70% night shifts	59.8	3.33
		> 70% day shifts	63.0	2.01
		Only day shifts	59.7	1.01
		Only night shifts	67.3	4.30
		A combination of day and night shifts	62.9	3.85
Weekend work	0.222	1 in 2	63.0	1.42
		1 in 3	60.6	1.90
		1 in 4	61.7	2.10
		1 day per weekend	61.0	2.29
		Infrequently (< 1 in 4)	55.4	2.62

		Never	56.3	2.91
Public holiday working requirement	0.019	Can choose	59.7	2.22
		Every public holiday	62.6	4.09
		Not required	58.7	1.20
		<50% of public holidays	60.8	1.95
		>50% of public holidays	66.6	1.97
Set roster pattern	0.017	No	63.9	1.55
		Yes	59.5	0.98
Timely roster notification	<0.001	No	70.3	2.37
		Yes	59.0	2.01
Average number of unpaid work hours per week	0.802	< 1	60.0	1.23
		1-3	60.2	1.73
		4-6	62.6	2.16
		7-10	60.9	3.63
		>10	62.5	2.73
Able to finish on time (workload)	<0.001	Never	71.8	2.77
		Rarely	64.1	1.62
		Occasionally	61.3	1.48
		Majority	56.7	1.32
		Always	47.7	4.26
Main reason for overtime	0.002	Scheduling	61.1	1.29
		Short staff	62.3	1.42
		Personal	42.1	4.84
		Walk-ins	61.7	1.89
		Other	57.9	3.24
Able to have meal break	<0.001	No	65.2	1.20
		Interrupted meal break	58.3	1.24
		Meal break without interruption	53.7	2.19
Adequate staffing in the past week	<0.001	No	64.8	0.99
		Yes	54.0	1.28
Workplace environment	<0.001	Supportive	57.3	0.95
		Toxic colleagues only	63.1	3.30
		Toxic	73.0	3.13
		Toxic management only	67.9	1.72
Bullying	<0.001	Experienced	64.7	1.85
		None	57.2	1.11
		Witnessed	64.7	1.53
Work satisfaction	<0.001	No	70.5	1.55
		Yes	57.5	0.89
Renumeration satisfaction	<0.001	No	64.5	1.12
		Yes	56.7	1.17
Socioeconomic status of clients	0.080	Affluent	64.8	2.71

		Diverse	61.8	1.46
		Low income	62.1	2.80
		Middle income	61.1	1.64
		Upper-middle	56.9	1.69
Frequency of interacting with adherent clients in the past week	0.004	Always	58.0	5.19
		Majority	59.7	0.90
		Occasionally	68.7	2.32
		Rarely/never	61.9	8.48
Frequency of encountering patient death in the past month	0.386	None	59.0	1.26
		1-3	62.4	1.27
		4-6	62.9	3.73
		> 6	59.2	3.73
		Do not recall	62.1	5.27
Frequency of euthanasia in the past month	0.150	None	61.6	4.29
		1-3	57.9	1.57
		4-6	60.5	1.44
		7-10	63.7	1.95
		> 10	62.8	2.02
Frequency of economic euthanasia in the past month	0.001	Frequently	66.7	3.22
		Occasionally	62.2	1.39
		Rarely	61.2	1.38
		Never	56.2	1.74
Frequency of delivering negative news in the past week	0.039	Always	67.3	3.46
		Frequently	62.4	1.19
		Occasionally	59.1	1.26
		Rarely/never	51.2	3.79
Frequency of interacting with emotionally distressed clients in the past week	<0.001	Always	66.8	2.63
		Frequently	66.1	1.24
		Occasionally	56.7	1.19
		Rarely/never	50.9	2.63

Supplementary Table 4. Univariable analysis results for Copenhagen Burnout Inventory – client-related burnout score. This table presents the P-value, predicted mean and standard error for each investigated factor and associated variable.

Factor	P value	Variables	Predicted mean	Standard error
Sex	0.030	Female	51.7	1.18
		Male	45.8	2.43
Age (years)	0.002	<30	56.0	2.75
		30-40	53.8	1.72
		40-50	47.7	2.11
		50+	45.2	2.11
Family composition	0.090	Married with dependent children	47.1	1.87
		Married with no dependents	50.9	1.68
		Single with dependent children	52.8	5.46
		Single with no dependents	54.1	2.11
Years of experience in principal practice	0.003	<5	55.4	2.01
		5 to 10	53.2	2.38
		10 to 20	49.2	2.11
		20+	45.5	1.99
Considering leaving principal practice in the past year	<0.001	No	42.0	1.56
		Yes	56.2	1.28
Position in practice	<0.001	Associate Veterinarian	53.2	1.17
		Management	48.6	3.52
		Owner	38.3	2.61
Hours worked per week	0.178	< 35 hours	51.0	1.89
		35-40 hours	51.8	1.81
		40-50 hours	49.8	2.20
		50-60 hours	51.6	3.86
		> 60 hours	38.1	5.25
Shift pattern	0.080	> 70% night shifts	56.7	4.22
		> 70% day shifts	50.4	2.54
		Only day shifts	49.3	1.28
		Only night shifts	62.8	5.44
		A combination of day and night shifts	50.8	4.87
Weekend work requirements	0.029	1 in 2	52.9	1.80
		1 in 3	54.8	2.40
		1 in 4	47.8	2.66
		1 day per weekend	50.5	2.90
		Infrequently (< 1 in 4)	43.5	3.32
		Never	45.0	3.68
Public holiday working requirement	0.650	Can choose	51.6	2.87
		Every public holiday	48.4	5.28

		Not required	50.1	1.56
		<50% of public holidays	48.4	2.52
		>50% of public holidays	53.5	2.55
Set roster pattern	0.213	No	52.7	1.98
		Yes	49.7	1.26
Timely roster notification	0.265	No	55.4	3.17
		Yes	50.7	2.69
Average number of unpaid work hours per week	0.054	< 1	52.6	1.55
		1-3	50.7	2.17
		4-6	51.0	2.73
		7-10	41.2	4.58
		>10	44.2	3.45
Able to finish on time (workload)	0.350	Never	49.2	3.73
		Rarely	50.9	2.18
		Occasionally	52.2	1.99
		Majority	50.3	1.77
		Always	39.8	5.73
Main reason for overtime	0.020	Scheduling	50.5	1.66
		Short staff	51.6	1.84
		Personal	31.0	6.25
		Walk ins	53.0	2.44
		Other	48.5	4.19
Able to have meal break	0.030	No	53.5	1.58
		Interrupted meal break	48.8	1.63
		Meal break without interruption	46.4	2.88
Adequate staffing	<0.001	No	53.3	1.32
		Yes	45.9	1.71
Workplace environment	0.180	Supportive	49.2	1.29
		Toxic colleagues only	49.5	4.47
		Toxic	56.9	4.24
		Toxic management only	53.4	2.33
Bullying	0.116	Experienced	53.1	2.42
		None	48.5	1.46
		Witnessed	52.7	2.00
Work satisfaction	<0.001	No	59.5	2.05
		Yes	47.6	1.18
Renumeration satisfaction	0.002	No	53.6	1.45
		Yes	47.0	1.51
Socioeconomic status of clients	0.118	Affluent	47.1	3.45
		Diverse	51.2	1.86
		Low income	52.1	3.57
		Middle income	54.1	2.09
		Upper-middle	46.7	2.15
Frequency of interacting with adherent clients in past week	<0.001	Always	31.8	6.49
		Majority	49.4	1.12

		Occasionally	62.4	2.90
		Rarely/never	48.6	10.59
Frequency of encountering patient death in the past month	0.860	None	50.1	1.61
		1-3	51.1	1.62
		4-6	51.3	4.77
		> 6	46.4	4.77
		Do not recall	54.2	6.75
Frequency of euthanasia in the past month	0.427	None	50.7	5.49
		1-3	57.7	2.00
		4-6	50.4	1.85
		7-10	53.6	2.50
		> 10	52.2	2.59
Frequency of economic euthanasia in the past month	<0.001	Frequently	56.0	4.00
		Occasionally	56.3	1.73
		Rarely	49.0	1.72
		Never	42.5	2.16
Frequency of delivering negative news in the past week	0.450	Always	56.7	4.48
		Frequently	51.0	1.54
		Occasionally	49.3	1.64
		Rarely/never	49.2	1.91
Frequency of interacting with emotionally distressed clients in the past week	<0.001	Always	58.8	3.46
		Frequently	55.3	1.63
		Occasionally	46.3	1.56
		Rarely/never	41.7	3.46

Supplementary Table 5. Univariable analysis results for Copenhagen Burnout Inventory total burnout score. This table presents the P-value, predicted mean and standard error for each investigated factor and associated variable.

Factor	P value	Variables	Predicted mean	Standard error
Sex	<0.001	Female	58.2	0.92
		Male	50.0	1.89
Age (years)	<0.001	<30	60.4	2.18
		30-40	59.8	1.36
		40-50	54.0	1.67
		50+	52.5	1.67
Family composition	0.081	Married with dependent children	54.4	1.49
		Married with no dependents	56.1	1.34
		Single with dependent children	58.7	4.33
		Single with no dependents	60.0	1.68
Years of experience in principal practice	0.001	<5	60.8	1.59
		5 to 10	58.6	1.89
		10 to 20	55.4	1.67
		20+	52.2	1.57
Considering leaving principal practice in the past year	<0.001	No	46.9	1.14
		Yes	63.1	0.93
Position in practice	<0.001	Associate Veterinarian	58.6	0.93
		Management	56.0	2.80
		Owner	47.0	2.08
Hours worked per week	0.620	< 35 hours	56.0	1.51
		35-40 hours	52.7	4.20
		40-50 hours	56.5	1.45
		50-60 hours	57.1	1.76
		> 60 hours	60.4	3.09
Shift pattern	0.098	> 70% night shifts	85.5	3.35
		> 70% day shifts	58.3	2.02
		Only day shifts	55.2	1.02
		Only night shifts	66.0	4.33
		A combination of day and night shifts	52.3	3.87
Weekend work requirements	0.063	1 in 2	59.1	1.43
		1 in 3	57.3	1.92
		1 in 4	55.8	2.12
		1 day per weekend	57.3	2.31
		Infrequently (< 1 in 4)	50.7	2.65
		Never	52.3	2.94
Public holiday working requirement	0.068	Can choose	55.8	2.26
		Every public holiday	59.8	4.16

		Not required	54.8	1.22
		<50% of public holidays	56.4	1.99
		>50% of public holidays	61.5	2.00
Set roster pattern	0.041	No	59.3	1.57
		Yes	55.5	0.99
Timely roster notification	0.001	No	65.3	2.39
		Yes	54.7	2.03
Average number of unpaid work hours per week	0.743	< 1	56.5	1.25
		1-3	56.1	1.75
		4-6	58.1	2.19
		7-10	52.6	3.68
		>10	57.8	2.77
Able to finish on time (workload)	<0.001	Never	64.6	2.88
		Rarely	59.1	1.68
		Occasionally	57.0	1.54
		Majority	53.9	1.37
		Always	43.8	4.43
Main reason for overtime	0.001	Scheduling	56.5	1.30
		Short staff	58.3	1.44
		Personal	36.7	4.89
		Walk-ins	57.9	1.91
		Other	55.3	3.28
Able to have meal break	<0.001	No	60.7	1.22
		Interrupted meal break	54.3	1.26
		Meal break without interruption	50.2	2.23
Adequate staffing	<0.001	No	60.2	1.02
		Yes	50.6	1.31
Workplace environment	<0.001	Supportive	53.4	0.98
		Toxic colleagues only	58.0	3.38
		Toxic	67.2	3.21
		Toxic management only	63.3	1.77
Bullying	<0.001	Experienced	61.2	1.89
		None	53.3	1.13
		Witnessed	59.7	1.55
Work satisfaction	<0.001	No	66.3	1.57
		Yes	53.4	0.91
Renumeration satisfaction	<0.001	No	60.5	1.13
		Yes	52.3	1.18
Socioeconomic status of clients	0.056	Affluent	59.0	2.73
		Diverse	58.1	1.48
		Low income	58.3	2.83
		Middle income	57.5	1.65
		Upper-middle	52.1	1.71
	<0.001	Always	49.0	5.20

Frequency of interacting with adherent clients in the past week		Majority	55.4	0.90
		Occasionally	66.0	2.33
		Rarely/never	56.1	8.49
Frequency of encountering patient death in the past month	0.417	None	55.1	1.27
		1-3	58.0	1.28
		4-6	59.4	3.78
		> 6	53.5	3.78
		Do not recall	58.2	5.34
Frequency of euthanasia in the past month	0.122	None	58.0	4.34
		1-3	53.3	1.57
		4-6	56.8	1.46
		7-10	59.3	1.97
		> 10	58.5	2.04
Frequency of economic euthanasia in the past month	<0.001	Frequently	62.8	3.22
		Occasionally	59.5	1.39
		Rarely	56.4	1.38
		Never	50.7	1.74
Frequency of delivering negative news in the past week	0.042	Always	61.8	3.53
		Frequently	58.0	1.21
		Occasionally	55.2	1.29
		Rarely/never	49.2	3.87
Frequency of interacting with emotionally distressed clients in the past week	<0.001	Always	62.7	2.66
		Frequently	62.0	1.26
		Occasionally	52.5	1.20
		Rarely/never	46.3	2.66
